# Perspectives of cellular communication through tunneling nanotubes in cancer cells and the connection to radiation effects

**DOI:** 10.1186/s13014-019-1416-8

**Published:** 2019-12-03

**Authors:** Nicole Matejka, Judith Reindl

**Affiliations:** Institut für angewandte Physik und Messtechnik, Universität der Bundeswehr München, Werner-Heisenberg-Weg 39, 85577 Neubiberg, Germany

**Keywords:** Cellular communication, Tunneling nanotubes, Radioresistance, Cancer

## Abstract

Direct cell-to-cell communication is crucial for the survival of cells in stressful situations such as during or after radiation exposure. This communication can lead to non-targeted effects, where non-treated or non-infected cells show effects induced by signal transduction from non-healthy cells or vice versa. In the last 15 years, tunneling nanotubes (TNTs) were identified as membrane connections between cells which facilitate the transfer of several cargoes and signals. TNTs were identified in various cell types and serve as promoter of treatment resistance e.g. in chemotherapy treatment of cancer. Here, we discuss our current understanding of how to differentiate tunneling nanotubes from other direct cellular connections and their role in the stress reaction of cellular networks. We also provide a perspective on how the capability of cells to form such networks is related to the ability to surpass stress and how this can be used to study radioresistance of cancer cells.

## Background

During cell survival and development, it is crucial for cells to have the possibility to communicate among each other. Without that essential tool they are not able to coordinate and organize themselves in complex cellular systems such as tissue or organisms [1]. Especially in stress situations which affect cell survival either directly through damaging DNA or indirectly through limiting the functionality of cellular organelles, communication plays a key role for the survival of a cell composite as already known since several decades [2, 3]. Moreover, the transfer of cellular organelles, proteins or signals from healthy to non-healthy cells can lead to enhanced cell survival capability [4–7]. Simultaneously, the same mechanisms can promote the progression of diseases such as Parkinson, Alzheimer, Huntington or HIV through transduction of viruses, bacteria and prions [5, 8–15]. Additionally, cellular communication plays a key role in different kinds of cancer, as it is e.g. known that the invasive potential and chemotherapy resistance is linked to enhanced communication activity in cancer cells [2, 16, 17] and also communication is altered in cancerous tissue [2]. The major effects, which are caused by cellular communication related to radiotherapy are non-targeted or Bystander effects [18, 19], where non irradiated cells show a radiation response which is expressed by e.g. genomic instability, enhanced apoptosis and enhanced DNA damage [20]. These responses have been attributed to direct transfer through gap junctions [21] or factors such as exosome-like vesicles [22], which are released by irradiated cells to their surroundings. The basic molecular mechanisms triggering these effects and especially how cellular communication plays a further role in the radiation induced enhancement of invasive and migrative potential of certain tumor types is widely unknown and a prominent target of current research.

In this context, cellular communication can be subdivided in two groups, contact and non-contact. The contact communication provides more rapid and diverse signal and molecule transfer compared to non-contact communication. Tunneling nanotubes (TNTs) represent a novel type of direct contact communication tool among cells [1]. TNTs are straight, thin membrane structures, connecting cells over long-distances and have been discovered by 3D live-cell microscopy in cultured rat pheochromocytoma PC12 cells in 2004 [23]. They appear as stretched branches between cells connecting these at their nearest distance above the substrate. After this discovery many similar findings in different cell lines were made [11, 24, 25] and a deluge of biological processes were reported in which TNTs could be involved [24, 26–28]. Upon this, TNTs were reported in healthy tissue including mouse heart [29] and mouse alveoli [30]. In the last 15 years, the research revealed a large diversity regarding morphology, composition and function of these membrane connections. It is generally agreed that they facilitate the direct cell-to-cell transfer of cargoes such as organelles, viruses and signals [8]. This mechanism enables cells to directly communicate with each other very quickly and effectively. There are several reviews covering the biology of TNTs in various cell lines [31–35].

Here, we focus on the role of TNTs in cancer cells and the connection to cellular reactions to stress, especially induced via radiation. As TNTs are more frequently formed at stress situations and in cancer cells especially in highly invasive cancer such as glioblastoma. This indicates that TNTs may play an important role in the direct cellular response to radiation. Therefore, we define TNTs as a prominent target for new approaches of glioblastoma therapy.

## Main text

### TNT definition

To date, a clear and totally agreed definition of TNTs does not exist. This is a consequence of numerous observations of similar structures which show on the one hand comparable but on the other hand different properties. However, some key characteristics can be satisfied about TNTs.

**Fig. 1:**
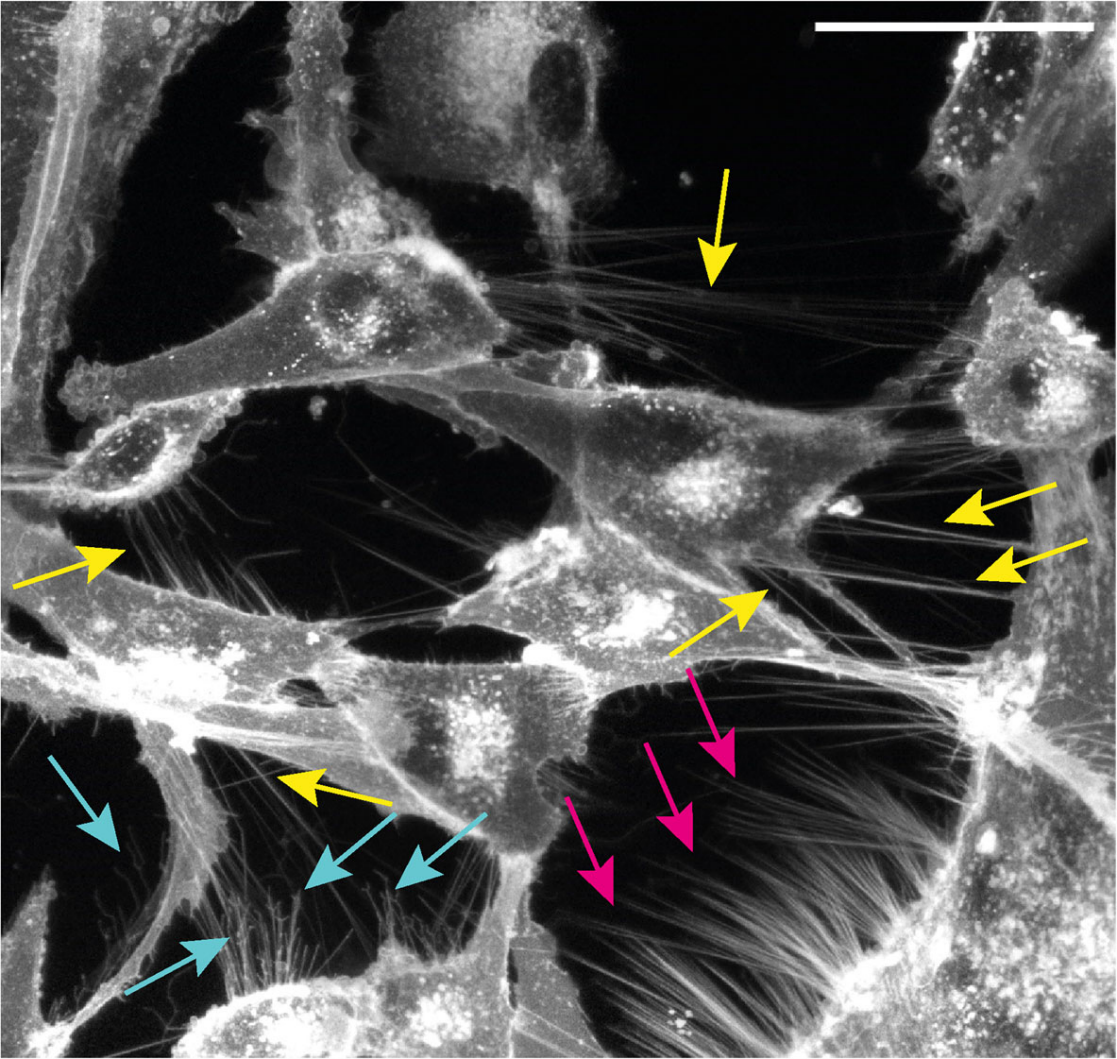
Confocal microscopy image of membrane labelled (CellMask© Orange) U87 glioblastoma cells. TNTs are the fine straight structures which interconnect cells, clear TNTs are marked by yellow arrows. Other fine structures which do not have cell-to-cell contact are filopodia (blue arrows). Magenta arrows point to structures which are not distinguishable between not yet fused TNTs and filopodia. U87 glioblastoma cells have a high frequency of TNTs. Scale bar 50 μm

**Fig. 2:**
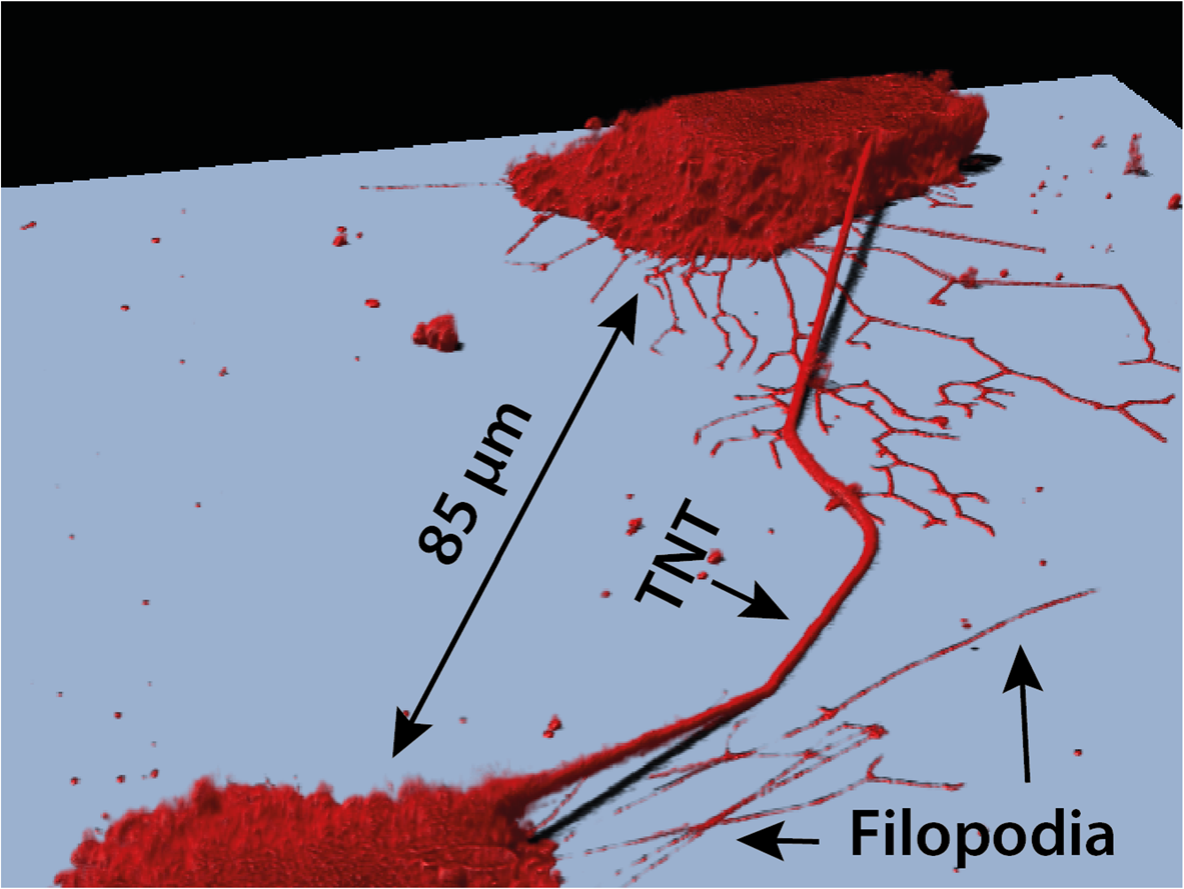
3D rendering of membrane labelled (PKH26) U87 glioblastoma cells interconnected by a special shaped TNT which has kinks and its middle section lies on the substrate. Filopodia can be clearly distinguished as they have no connections

**Fig. 3:**
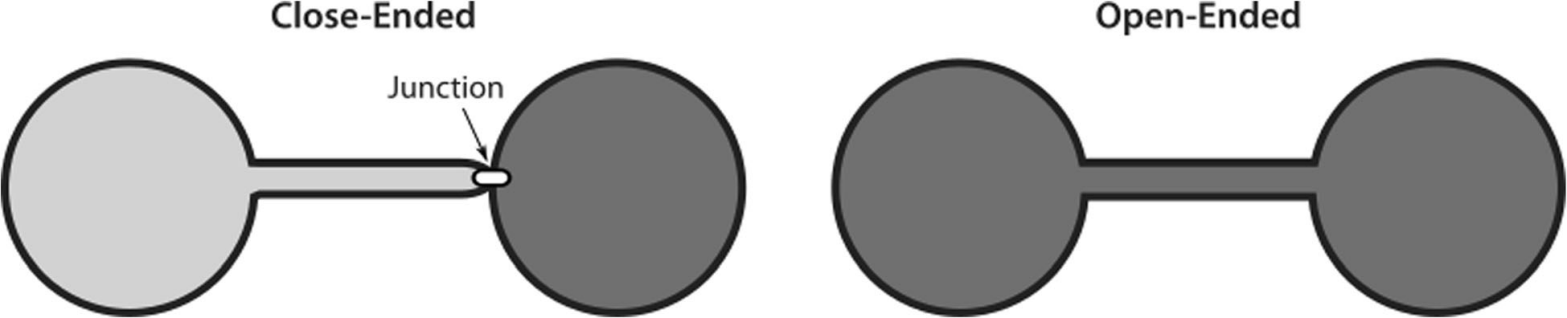
Schematic representation of the two reported kinds of TNT connection to the cell body. On the left side a close-ended TNT is illustrated. Instead of membrane continuity there is a distinct junction between the connected cells recognizable. Such a junction is mostly found at one end of the nanotube as drawn here, but it has been also observed that these junctions can dynamically shift along the membrane tunnel. On the right side an open-ended nanotube is drawn, there the membrane of the tunnel has been fused with the plasma membrane of the connected cell and thus a membrane continuity was generated

TNTs are thin cytoplasmic membrane bridges with a diameter ranging from 50 nm to 1500 nm that interconnect cells over long distances up to several cell diameters length [8] (see Fig. 1). This allows the direct cell-to-cell transfer of signals as well as cellular compounds [8, 24]. They often appear as straight lines in-vitro, but in tissue or in three dimensional extracellular matrix they can exhibit a curved morphology [11, 25, 36]. In Fig. 2 a 3D rendering of a TNT connection between U87 glioblastoma cells is shown which has kinks and its middle part lies on the substrate. Due to their flexible shape, TNTs are also able to connect cells even if the nearest distance between them is blocked e.g. by other cells [11]. Between cell lines or even in the same cell line the morphology and cytoskeletal composition of TNTs vary [24]. Whereas F-actin is found in most TNTs, usually only the thicker TNTs contain microtubules [25] or cytokeratin filaments [37]. The length varies in a range of just a few to over 100 μm [11] and can be dynamically regulated if the interconnected cells migrate until the distance becomes too large and the tube disappears [24]. The lifetime is ranging from a few minutes [11, 38] up to several hours [12, 26]. Both length and life time might be determined by the available membrane reservoirs and migration speed [26, 39]. Additionally, TNTs which contain microtubules may be more stable than those composed only of F-actin since microtubule-filaments exhibit a higher degree of stiffness [24]. Besides the differences regarding the cytoskeleton content, also the connection of a TNT to the cell body varies among different cell types. Some TNTs are open-ended at both ends and thus exhibit membrane continuity [23, 40], but there are also close-ended TNTs containing a junction [11, 41, 42] or immune synapse [43] as gating mechanism. A sketch of open- and close-ended TNTs is displayed in Fig. 3.

TNTs have to be distinguished from other similar membrane structures such as cytonemes [44, 45] and filopodia [46] (see Fig. 1 and Fig. 2), which do not have cell-to-cell contact. Additionally, there are membrane connections which also allow cell-to-cell communication, but are structurally distinct from TNTs, the gap junctions [47] and epithilial bridges [48]. The key features of these connections and protrusions are shown in Table 1.

**Table 1:** Overview and comparison between intercellular connections and membrane protrusions similar to TNTs

Name	Diameter	Length	Cell-to-cell contact	Cytosceletal content	Positioning in-vitro	References
TNT	50–1500 nm	Few to over 100 μm	Yes	F-actin, some microtubules, some cytokeratin filaments	Usually above substrate	[8, 24]
Cytoneme	< 200 nm	Up to 70 μm	No	F-actin, no microtubules	N/A, tissue only	[44, 45]
Filopodia	100–300 nm	Several μm	No	F-actin, no microtubules	Contact to substrate	[46]
Gap junctions	< 10 nm	~ 10 nm	Yes	N/A	Above substrate	[47]
Epithilial bridges	1–20 μm	25–100 μm	Yes	F-actin, microtubules	Above stubstrate	[48]

### TNT formation

**Fig. 4:**
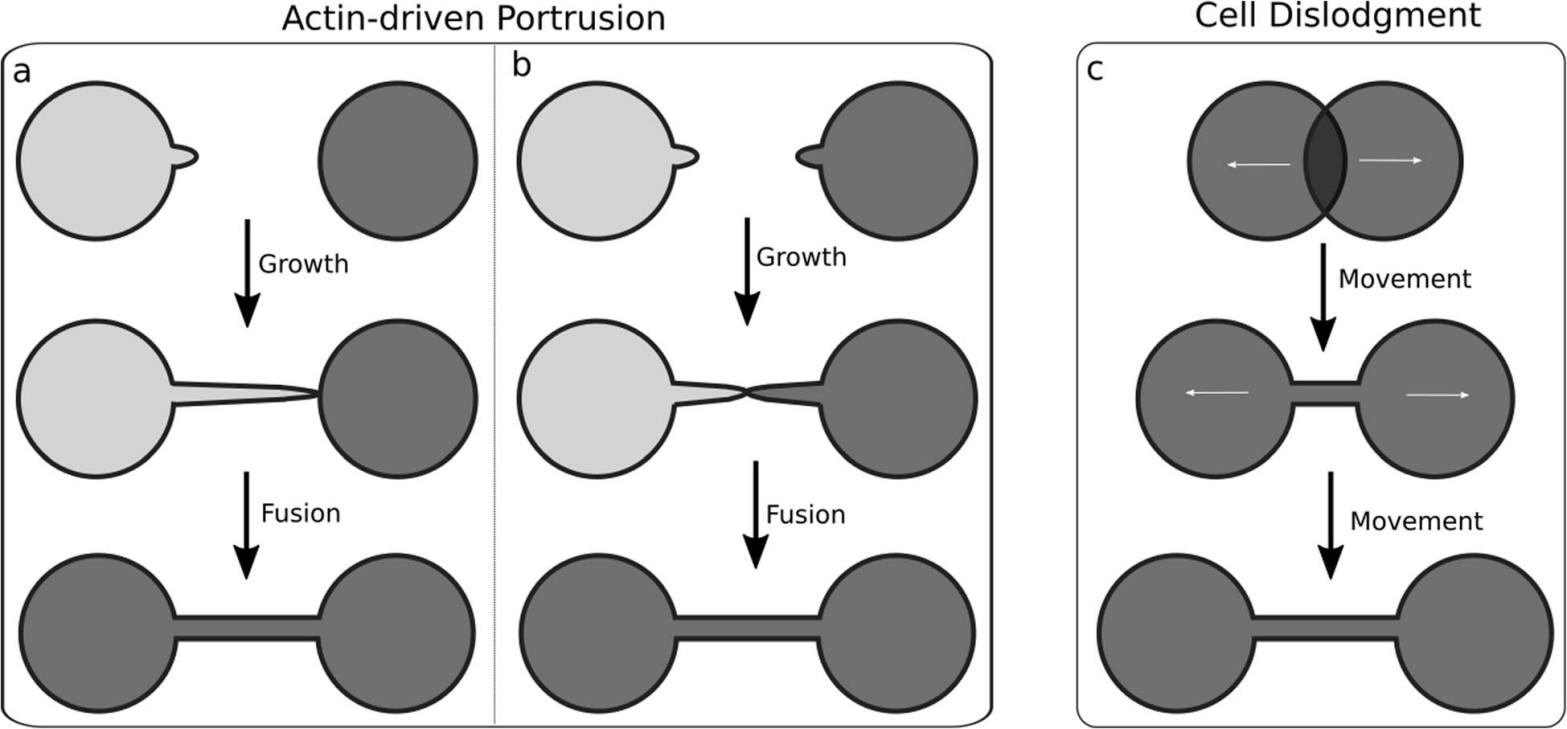
Illustrations of TNT formation models. On the left side, the formation of a nanotube by the actin-driven growth of membrane protrusions is shown. **a**) The protrusion from one cell elongates until it reaches the target cell, where physical contact will be established by adhesion followed by a membrane fusion of tunnel and target cell. An open-ended nanotube connection will be generated. **b**) It might also be possible that two different membrane protrusions meet each other and establish a connection by adhesion and fusion. **c**) On the right side, the second formation model is illustrated, the TNT formation by cell dislodgement. Here, the cells migrate apart from each other after physical contact and during their migration the nanotunnel will be pulled out of the cells. At the end of the migration, the cells are still connected via the generated TNT

Based on the observations of TNT formation in research two ways of TNT establishment were identified [49]. The first is the de novo generation of nanotubes from filopodia-like protrusions by an actin-driven process within several minutes [10, 23, 38] (Fig. 4 left side). The main differentiation between filopodia and not yet fused TNTs in this case is, that filopodia seem to be more branched and with contact to the surface in cell culture, which TNTs normally don’t have. But a clear distinction is difficult or even impossible (see Fig. 1), which gives an additional hint on the complexity of the function of cellular membrane protrusions. Here, a protrusion of membrane, probably initiated by Rho-family GTPases, elongates by actin polymerization. If the tip of the protrusion reaches the target cell or another protrusion, a physical contact will be establish by adhesion and possibly membrane fusion [23] (Fig. 4 left side). However, it is uncertain whether the filopodia growth is a stochastic process or driven by a chemoattractant.

The second way is the TNT formation at the detachment of cells after direct cell-to-cell contact (Fig. 4 right side). This mechanism has been observed in immune cells as T cells, natural killer cells or macrophages as well as in normal rat kidney cells [11, 12, 41–43]. Here, cells form an immune synapse or fuse when they come into direct contact. With the dislodgement of the cells a nanotube is pulled out (Fig. 4 c). Whether one or both cells contribute to the establishment of the TNT is uncertain. Additionally, the involvement of adhesion, fusion or actin polymerization remains unknown in this formation mechanism [49]. The time of cell-to-cell contact seems to play a key role, since TNTs are only hardly established if the cell-to-cell contact lasts under 4 min in T cells [11, 43]. Even though, these two models might appear to be very distinct, they are not mutually exclusive [49, 50]. Several stimuli of TNT formation such as Fas-ligand receptor in the immune system or M-sec, a protein associated with the component Sec6 of the exocyst complex required for the docking of exocytic vesicles on the plasma membrane [51, 52], have been discovered. These findings suggest that the TNT formation might be like the mechanisms of filopodia and lamelipodia regulation. Involved molecules in the TNT formation machinery are Rho and Ras small GTPases families such as Cdc42, Ral or the exocyst effector [52] as well as myosin X [49] and the transmembrane MHC class III protein LST1 [53]. These molecules are responsible for the actin cytoskeleton remodelling along the protrusion steps of TNT formation. Furthermore, lipid draft proteins such as I-Bar [54] which generate cell membrane curvature are required for TNT stabilization and formation. For TNT guidance and initiation of cell-to-cell contact, adhesion molecules such as N-cadherin and β–catenin [37, 55] as well as receptor-ligand interactions [43] are crucial. For more detailed research on the molecular basis of TNT formation and the inception mechanisms in the TNT development please refer to [31, 34, 56].

### Exchange of organelles and particles

TNTs can be used as highways to transfer and exchange cellular compounds from one cell to another. Organelles and particles, which are observed to be interchanged between cells are mitochondria [4–6, 12, 16, 29, 57–60], vesicles [12, 23, 37, 38], membrane as well as cytoplasmic components [12, 23, 40, 51], nanoparticles [61–64] and even more [17, 28, 49, 57, 65]. Furthermore, the research on TNTs pointed out that there are different transport mechanisms which may be dependent on the cytoskeletal content, diameter as well as membrane continuity of the considered nanotube. For instance, in human macrophages two different types of TNTs are found. Here, intracellular components including endosomes, lysosomes and mitochondria are only exchanged within thick, microtubulin containing TNTs, whereas surfing of bacteria on the membrane surface is only detectable in thin actin-based TNTs [12].

### Signal transfer

It has been proven that TNTs have a prominent role in the propagation of signals. For instance, Ca^2+^ signals can be transferred via TNTs between remote cells [29, 40–42, 52, 66]. Usually, electrical signals are transmitted through neuronal synapses or gap junctions, latter need a close proximity for cell-to-cell communication. In contrast, these kinds of signals can be quickly transferred over long distances when cells are connected by TNTs. That phenomenon was initially reported in 2005 [40]. Treatment with α-glycyrrhetinic acid, an inhibitor for the functionality of gap junctions, does not block the Ca^2+^ flux transfer, suggesting that gap junctions are not involved in this transmission. Recent studies using the sensitive membrane potential probe DiBAC4 [3] reveal that several cell types can be electrically coupled by TNTs, where gap junctions interposed at the membrane interface in one end of the connection allowing bi-directional passage of electrical currents in a selective way [41, 42]. The strength of signal depends on the length and diameter of the nanotubes, the open probability of present gap junctions as well as the number of involved TNTs per connection. The fact, that there are both gap junction independent and gap junction dependent electrical transmissions by TNTs implies a significant diversity of TNTs with different properties and functions.

Besides electrical signals, death signals can also be transferred through TNTs [43, 51]. The involvement of TNTs in apoptosis signalling was found in T cells, in which death signals are propagated by TNTs and immune stimulation leads to an enhanced TNT formation among cells. In fact, it was shown that phagocytosis signals are transferred from apoptotic to viable cells in order to help the immune system to detect a damaged cellular region [67]. Furthermore, it was reported that activated natural killer cells more frequently form TNTs and connected target cells are more often lysed than unconnected [43]. This suggests that TNTs can help the immune system to transfer cytotoxic chemicals to distant target cells.

### Transport mechanisms

The research on TNTs uncovers a significant diversity of transport processes. One observed and suspected transport mechanism is the exchange of molecules by molecular motors [23, 27] as several transferred molecules and particles such as HIV-1 viral particles or lysosomal vesicles exhibit a velocity in a range similar to those of actin-driven molecular motors [49]. Furthermore, this myosin-driven transport mechanism is in the agreement with the unidirectional one-way street feature, since this could be established by assuming that the actin filaments are of the same polarity [68]. It could also be possible, that the cargoes are linked to actin themselves and transported by actin polymerization [23]. Here, the actin could be seen as a rope on which the cargoes are anchored and dragged along the tube driven by actin polymerization at one end [27]. Bidirectional transport of cargoes was only observed in TNTs which additionally contain microtubules as cytoskeletal content suggesting that a microtubule molecular motor could be responsible for this behaviour [49]. It was also observed that bidirectional transfer of cargoes can change into an unidirectional transport proceeding after stress situations such as injury [28]. It might also be possible, that there are transport mechanisms which are cytoskeleton independent such as the transfer by gondolas [38]. Gondolas, are moving distensions in TNTs which can carry enclosed organelles which are bigger than the diameter of the respective nanotube itself [69]. The formation and the generating force needed for their movement are unexplored. It is possible that the movement is driven by differences in chemical potential regarding to the molecules inside the bulk solution and the interior of the target cell or to the compositions of the gondola membrane and the target cell membrane [37]. Further investigations must be performed in order to resolve how the carriage of cargoes occurs within TNTs and which molecular motors or other proteins are involved in such a transfer system. Nevertheless, many reports demonstrate that the transfer of molecules and particles occurs in an active manner and not due to diffusion. For instance, studies with ATP reveal a blockage of organelle transfer via nanotubes pointing out that ATP is necessary for the respective transferring processes [10, 12, 26, 28]. Same results were obtained by considering the transfer of electrical signals across TNTs, here simulations showed that a passive transfer is inefficient and experiments indicate that the signals are actively generated and propagated within TNTs [66].

### Relation to stress

Several studies point out that the presence of TNTs as cellular network correlates with diverse stress factors such as hydrogen peroxide (H_2_O_2_) exposure [70], hypoxia [71], UV [4], x-ray [72] and particle radiation [73] as well as serum starvation [59], temperature, toxin B [54], infections [14, 74] or inflammation [36, 39, 56]. Furthermore, it is revealed that healthy cells are able to rescue apoptotic cells by the exchange of functional mitochondria via TNTs [4, 5]. These findings suggest that TNTs have a special relation to stress. In his recent paper, Amin Rustom used these findings to introduce a new mechanistic model of reactive oxygen species-dependent tunneling nanotube formation [75]. He describes the formation of TNTs according to the increase of reactive oxygen species (ROS) level in stressed cells. These stressed cells transmit “call-for-help” signals to their surroundings. According to this model, TNTs will be formed by unstressed cells in order to establish an open communication channel to the stressed cell. Followed by the exchange of particles such as mitochondria to rescue the apoptotic cell or by the isolation and removal of the cells whose ROS level is too excessive. Based on this model, TNTs are a communication tool used for the cellular organization and survival during stress.

However, the exact role of TNTs in stress situations remains obscure. Although, more and more reports of TNTs under stressful conditions become published, it is still unclear under which circumstances the TNTs are established from the non-stressed to the stressed cells as in the model of Rustom [75] or vice versa as reported in other studies [4, 76]. Wang et al. [76] found that the transcription factor p53 which regulates several genes in response to various harmful stress signals including DNA damage and hypoxia [77], plays an important role for the development of TNTs in astrocytes after H_2_O_2_ treatment and serum depletion. Nevertheless, it is still unknown which mechanisms or signals activate the cells to form an open communication channel and thus respond to stress. Additionally, the role of stress proteins such as heat shock proteins in the formation of TNTs is widely unexplored. A recent study showed that the membrane-bound heat shock protein mHsp70 is located on TNTs in human U87 glioblastoma, mouse GL261 glioma and mouse 4 T1 mammary carcinoma cell lines [73]. This finding provides the first evidence of the presence of stress proteins as structural component in the lipid composition of TNTs and therefore the support of TNTs by stress proteins.

### Connection to cancer

TNTs have been found in several cancer cell lines including glioblastoma [72, 73, 78–80], carcinomas [38, 62, 63, 73], ovarian cancer [57, 71, 81–84], breast cancer [17, 57, 59, 81, 82], bladder cancer [16, 54], HeLa [15, 52], human neuroblastoma [17] and mesothelioma cell lines [58, 59, 85–87]. Furthermore, Ady et al. [85] showed that malignant mesothelioma cells exhibit 20-fold to 80-fold more TNTs as compared to normal mesothelial cells after 72 h in vitro cell culture. Thus, TNTs are a preferred communication mechanism in malignant cell growth. In addition, TNTs are not only found in in vitro cancer cell cultures but also in vivo. The first evidence for the existence of TNTs in vivo was provided by Chinnery and colleagues in 2008 [36]. They showed that MHC class II^+^ cells in the mouse corneal stroma are interconnected by TNTs and that this network is more distinct under inflammatory conditions. In 2012, Lou et al. [59] demonstrate the occurrence of TNTs in solid tumor samples from patients with mesothelioma and lung adenocarcinoma. TNTs were also found in tumor explants of murine orthotopic osteosarcoma and human patients with ovarian cancer [57, 71, 83]. Osswald et al. [72] discovered that membrane tubes are jointly responsible for the high resistance and progression of brain tumors when acting as a functional multicellular network.

This statement becomes even clearer when considering the so far observed exchanges of different cargoes via TNTs in cancer cells. It has been reported, that mitochondria traffic through TNTs supports the invasiveness of bladder cancer cells or modulates chemoresistance [16, 57]. Furthermore, TNTs are also suspected to transfer P-glycoproteins in cancer cells, a protein which can cause multidrug resistance [17, 82]. Chemoresistance can also be accomplished by the transfer of genetic material such as microRNA through TNTs [83, 88]. Although, cellular communication via TNTs can cause redistribution of drugs which in turn leads to increased resistance to chemotherapy or radiotherapy, the opposite can also be true. A recent study on the bystander effect showed that the herpes simplex virus NV1066 can be delivered to non-infected recipient cells by TNTs [86]. Here, the authors used a modified transwell assay that physically separates the cells by a 0.4 μm thick membrane to prevent contact-dependent cellular communication via gap junctions and to reduce the exchange of diffusing exosomes by > 80% [89]. This finding demonstrates that cell-to-cell communication via TNTs can induce apoptosis in non-targeted cells and thus may also be a promising tool to enhance the effectivity of a therapeutic treatment.

### TNTs and radiotherapy

Radiotherapy is one of the four pillars in cancer therapy as approx. 50% of all tumors worldwide are treated using radiotherapy [90, 91]. In particular, patients with central nervous system, breast, oesophageal, lung, head and neck cancers frequently receive radiotherapy during their course of illness [90]. Although less is known about the role of TNTs after irradiation first studies point on the one hand to a protecting effect of TNTs after x-ray radiation [72] and on the other hand to a decrease of connections after alpha-particle irradiation [73]. This points to a versatile role of TNTs in the radiation response of a cell composite. Additionally, TNTs were found in several cell lines which originate from the tumor types which are treated using radiotherapy. Furthermore, tumors such as glioblastoma which are well known to be highly migrative and invasive [92] show low response to treatment resulting in low 5-year survival rate [93, 94]. Moreover the invasive potential of these tumors is enhanced upon conventional radiation treatment using x-rays [95, 96] but not after alpha-particle treatment [95]. Additionally, it is well known that photon radiation induces cellular stress via the induction of ROS such as hydrogen peroxide (H_2_O_2_) which are suspected for triggering TNT formation [70, 75]. Particle radiation on the other hand predominantly directly interacts with the DNA and therefore ROS induction might be reduced [97].

## Conclusion

In this short review, we report on intercellular communication via tunnelling nanotubes. These membrane connections can be especially characterized by their dimensions, which are 50 nm to 1500 nm in diameter and their length can dynamically be regulated from a few microns to over 100 μm. They facilitate fast and direct signal as well as organelle transfer between distant cells. By establishing multicellular functional networks both cell progression and stress response but also pathogen spreading can be improved. We show that although many reports about TNTs in various cell types and situations were published, there is little known about actual working proceeding of the transport mechanisms seen in TNTs. Furthermore, the triggering and regulation of their formation or stability and their connection to the cell body are widely unknown principles.

Nevertheless, versatile features of TNTs are already found to play important roles in various fields of disease and especially cancer research. Key roles are reported in the immune response system, cell development, repair and survival, cancer progression as well as in the spreading of pathogens such as bacteria, viruses or misfolded proteins. This opens the opportunity to speculate about their potential as promising therapeutic target [98, 99]. For example, on the one hand the formation of TNTs can actively be blocked in order to interrupt the spreading of pathogens and to inhibit the TNT-mediated high therapeutic resistance. Especially drugs targeting the polymerization of actin or even triggering its depolymerization such as Cytochalasin B and Lactrunculin B [71] seem to be promising. On the other hand, TNTs can be used as cellular highways for drug delivery [8]. TNTs may therefore open up new possibilities for the diffusion or selective transport of therapeutics or cellular organelles inside the communication system of desired target cells [28, 64]. The above results also show that cellular communication via TNTs not solely occurs in the test tube, instead it is also tissue relevant and consequently plays a role in the fight against cancer. For example, the exchange of drugs might be a target to be investigated for the selective radio sensitization of tumor cells, as through the enhanced TNT formation the drug delivery might be faster compared to normal tissue.

In this review, we focused especially on the relation of TNTs to stress and their connection to cancer in order to work out their potential for their usage in relation to radiotherapy. Certain occurrence of TNTs under stress conditions such as hypoxia [71] which is a characteristic stress situation in the tumor microenvironment or H_2_O_2_ induction as well as the occurrence in tumor types which are treated with radiotherapy and especially the ones with bad prognosis, implies the importance of TNTs in the alarm system of cancer cells. Due to this distinct stress response, the survival rate of cancer cells may be potentially increased the more cells are able to form TNTs and the more stress signals are spread in the surrounding. Taking this together, TNTs should be considered as a reason for the low efficiency of conventional radiotherapy in e.g. highly invasive glioblastoma cells.

A further feature of the cellular communication via TNTs is the facility to exchange cargoes over long distances in a very direct and selective manner. In the tumor microenvironment, there is a great heterogeneity in the composition of several kinds of cells including stromal, tumor, red blood cells and so on [79, 100]. Due to this high complexity in the cellular matrix of a tumor region, non-contact cellular communication by secretion of signal molecules or contact cellular communication by gap junctions which require an immediate proximity of donor and recipient cell, are very limited and the flexible TNTs connections may present a more promising opportunity for cells to communicate and network among each other.

Furthermore, the occurrence of effects on non-treated cells called non-targeted or Bystander effects are well known in radiation treatment of cancer cells [18, 19]. The underlying mechanisms behind these are not yet clear. It is speculated that stress molecules are transferred via exosomes or direct connections such as gap junctions. However, the role of TNTs in the non-targeted effects is up to now poorly studied and not yet known.

**Fig. 5:**
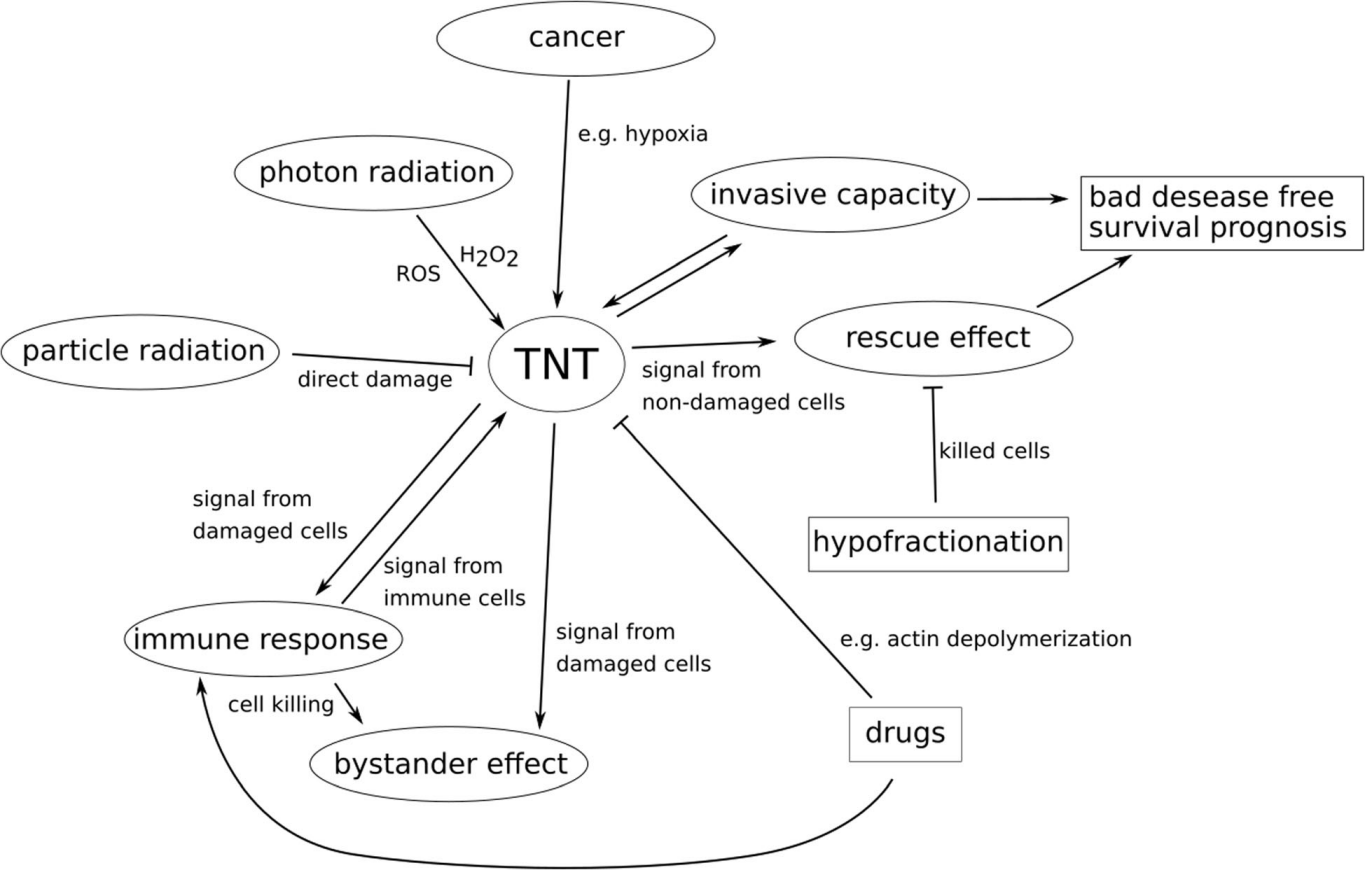
Mind map summarizing the complex interactions of TNTs related to radiotherapy

Taken altogether, it is indisputable that TNTs are strongly linked to cancer and therefore might also be a potential target for new therapeutic approaches. Especially when considering radiotherapy this seems to be promising for two reasons. New innovative radiotherapy approaches using particles such as protons and carbon ions are used to enhance normal tissue protection by keeping tumor control or even enhancing this [101, 102]. Due to the dose distribution of particles in tissue following the Bragg peak, dose is decreased in the heathy tissue whereas kept in the tumor [103]. Furthermore, these therapy approaches rely on more efficient cell killing by directly interacting with the DNA rather than inducing ROS. Taking the two things together this opens the possibility for hypofractionation [104, 105], which would dramatically decrease the time used for tumor treatment. This would consequently lower the time for the tumor cells to form TNT networks and therefore be rescued. On the other hand, the research on TNT based rescue effects makes it even more important for treatment to kill all tumor cells, as the damaged but surviving cells might be healed by the surrounding tissue. Furthermore, there are hints in the literature [63, 64] that particle radiation in contrast to photon radiation lowers the amount of TNTs in glioblastoma. Therefore considering the usage of particles rather than photons for the therapy of certain tumors would further decrease the capability of the cancer cells to circumvent death through radiotherapy. In Fig. 5 we summarize the various effects of TNTs and on TNTs which can occur during radiotherapy. The complex interconnection of effects related to TNTs which are illustrated here show the importance of TNTs in the cellular response to radiation. We also want to point out that studying the not yet known molecular mechanisms, by which radiation triggers TNT formation or disruption needs to be exploited as well in order to be able to choose the right radiation therapy method for each individual tumor. We therefore conclude that TNTs are a crucial target which has to be investigated in order to understand therapy outcome and to be able to find new and more effective tumor treatment. This opinion is shared by many researchers and in the last few years several reviews covering the role of TNTs in cancer as well as the paradigm to exploit intercellular communication to better treat cancer have been published [58, 79, 80, 100, 106–108].

## Data Availability

Not applicable.

## Abbreviation

TNT: Tunneling nanotube ROS reactive oxygen species H_2_O_2_ hydrogen peroxide

## References

[1] Buszczak M, Inaba M, Yamashita YM (2016). Signaling by cellular protrusions: keeping the conversation private. Trends Cell Biol.

[2] LOEWENSTEIN W. R., KANNO Y. (1966). Intercellular Communication and the Control of Tissue Growth: Lack of Communication between Cancer Cells. Nature.

[3] Borek Carmia, Higashino S., Loewenstein W. R. (1969). Intercellular communication and tissue growth. The Journal of Membrane Biology.

[4] Wang X, Gerdes H-H (2015). Transfer of mitochondria via tunneling nanotubes rescues apoptotic PC12 cells. Cell Death Differ.

[5] Guo R, Davis D, Fang Y (2018). Intercellular transfer of mitochondria rescues virus-induced cell death but facilitates cell-to-cell spreading of porcine reproductive and respiratory syndrome virus. Virology.

[6] Rodriguez A-M, Nakhle J, Griessinger E, Vignais M-L (2018). Intercellular mitochondria trafficking highlighting the dual role of mesenchymal stem cells as both sensors and rescuers of tissue injury. Cell Cycle.

[7] Torralba D, Baixauli F, Sánchez-Madrid F (2016). Mitochondria know no boundaries: mechanisms and functions of intercellular mitochondrial transfer. Front Cell Dev Biol.

[8] Ariazi J, Benowitz A, de Biasi V, Den Boer ML, Cherqui S, Cui H (2017). Tunneling nanotubes and gap junctions-their role in long-range intercellular communication during development, health, and disease conditions. Front Mol Neurosci.

[9] Gousset K, Schiff E, Langevin C, Marijanovic Z, Caputo A (2009). Browman DT et al. Prions hijack tunnelling nanotubes for intercellular spread ncb.

[10] Gousset K, Zurzolo C (2009). Tunnelling nanotubes: a highway for prion spreading?. Prion.

[11] Sowinski Stefanie, Jolly Clare, Berninghausen Otto, Purbhoo Marco A., Chauveau Anne, Köhler Karsten, Oddos Stephane, Eissmann Philipp, Brodsky Frances M., Hopkins Colin, Önfelt Björn, Sattentau Quentin, Davis Daniel M. (2008). Membrane nanotubes physically connect T cells over long distances presenting a novel route for HIV-1 transmission. Nature Cell Biology.

[12] Onfelt B, Nedvetzki S, Benninger RKP, Purbhoo MA, Sowinski S, Hume AN (2006). Structurally distinct membrane nanotubes between human macrophages support long-distance vesicular traffic or surfing of bacteria. J Immunol.

[13] Hurtig J, Orwar O (2008). Injection and transport of bacteria in nanotube–vesicle networks. Soft Matter.

[14] Sherer NM, Lehmann MJ, Jimenez-Soto LF, Horensavitz C, Pypaert M, Mothes W (2007). Retroviruses can establish filopodial bridges for efficient cell-to-cell transmission. Nat Cell Biol.

[15] Abounit S, Wu JW, Duff K, Victoria GS, Zurzolo C (2016). Tunneling nanotubes: a possible highway in the spreading of tau and other prion-like proteins in neurodegenerative diseases. Prion.

[16] Lu J, Zheng X, Li F, Yu Y, Chen Z, Liu Z (2017). Tunneling nanotubes promote intercellular mitochondria transfer followed by increased invasiveness in bladder cancer cells. Oncotarget.

[17] Levchenko A, Mehta BM, Niu X, Kang G, Villafania L, Way D (2005). Intercellular transfer of P-glycoprotein mediates acquired multidrug resistance in tumor cells. Proc Natl Acad Sci U S A.

[18] Desouky O, Ding N, Zhou G (2015). Targeted and non-targeted effects of ionizing radiation. Journal of Radiation Research and Applied Sciences.

[19] Prise KM, O'Sullivan JM (2009). Radiation-induced bystander signalling in cancer therapy. Nat Rev Cancer.

[20] Yahyapour R, Motevaseli E, Rezaeyan A, Abdollahi H, Farhood B, Cheki M (2018). Mechanisms of radiation bystander and non-targeted effects: implications to radiation carcinogenesis and radiotherapy. Curr Radiopharm.

[21] Marín A, Martín M, Liñán O, Alvarenga F, López M, Fernández L, et al. Bystander effects and radiotherapy. Reports of Practical Oncology & Radiotherapy 2015; 20(1):12–21. Available from: URL. http://www.sciencedirect.com/science/article/pii/S1507136714001424. 10.1016/j.rpor.2014.08.004 PMC4268598 25535579

[22] Ariyoshi K, Miura T, Kasai K, Fujishima Y, Nakata A, Yoshida M. Radiation-induced bystander effect is mediated by mitochondrial DNA in exosome-like vesicles. Sci rep; 9(1):1–14. Available from: URL. https://www.nature.com/articles/s41598-019-45669-z.pdf. 10.1038/s41598-019-45669-z PMC6591216 31235776

[23] Rustom A, Saffrich R, Markovic I, Walther P, Gerdes H-H (2004). Nanotubular highways for intercellular organelle transport. Science.

[24] Austefjord MW, Gerdes H-H, Wang X (2014). Tunneling nanotubes: diversity in morphology and structure. Commun Integr Biol.

[25] Seyed-Razavi Y, Hickey MJ, Kuffová L, McMenamin PG, Chinnery HR (2013). Membrane nanotubes in myeloid cells in the adult mouse cornea represent a novel mode of immune cell interaction. Immunol Cell Biol.

[26] Gurke S, Barroso JFV, Gerdes H-H (2008). The art of cellular communication: tunneling nanotubes bridge the divide. Histochem Cell Biol.

[27] Gerdes H-H, Bukoreshtliev NV, Barroso JFV (2007). Tunneling nanotubes: a new route for the exchange of components between animal cells. FEBS Lett.

[28] Marzo L, Gousset K, Zurzolo C (2012). Multifaceted roles of tunneling nanotubes in intercellular communication. Front Physiol.

[29] He K, Shi X, Zhang X, Dang S, Ma X, Liu F (2011). Long-distance intercellular connectivity between cardiomyocytes and cardiofibroblasts mediated by membrane nanotubes. Cardiovasc Res.

[30] Islam MN, Das SR, Emin MT, Wei M, Sun L, Westphalen K (2012). Mitochondrial transfer from bone-marrow-derived stromal cells to pulmonary alveoli protects against acute lung injury. Nat Med.

[31] Sisakhtnezhad S, Khosravi L (2015). Emerging physiological and pathological implications of tunneling nanotubes formation between cells. Eur J Cell Biol.

[32] McCoy-Simandle K, Hanna SJ, Cox D (2016). Exosomes and nanotubes: control of immune cell communication. Int J Biochem Cell Biol.

[33] Zaccard CR, Rinaldo CR, Mailliard RB (2016). Linked in: immunologic membrane nanotube networks. J Leukoc Biol.

[34] Drab Mitja, Stopar David, Kralj-Iglič Veronika, Iglič Aleš (2019). Inception Mechanisms of Tunneling Nanotubes. Cells.

[35] Murray Lisa M.A., Krasnodembskaya Anna D. (2018). Concise Review: Intercellular Communication Via Organelle Transfer in the Biology and Therapeutic Applications of Stem Cells. STEM CELLS.

[36] Chinnery HR, Pearlman E, McMenamin PG (2008). Cutting edge: membrane nanotubes in vivo: a feature of MHC class II+ cells in the mouse cornea. J Immunol.

[37] Veranic P, Lokar M, Schütz GJ, Weghuber J, Wieser S, Hägerstrand H (2008). Different types of cell-to-cell connections mediated by nanotubular structures. Biophys J.

[38] Bukoreshtliev NV, Wang X, Hodneland E, Gurke S, Barroso JFV, Gerdes H-H (2009). Selective block of tunneling nanotube (TNT) formation inhibits intercellular organelle transfer between PC12 cells. FEBS Lett.

[39] Ranzinger J, Rustom A, Abel M, Leyh J, Kihm L, Witkowski M (2011). Nanotube action between human mesothelial cells reveals novel aspects of inflammatory responses. PLoS One.

[40] Watkins SC, Salter RD (2005). Functional connectivity between immune cells mediated by tunneling nanotubules. Immunity.

[41] Wang X, Gerdes H-H (2012). Long-distance electrical coupling via tunneling nanotubes. Biochim Biophys Acta.

[42] Wang X, Veruki ML, Bukoreshtliev NV, Hartveit E, Gerdes H-H (2010). Animal cells connected by nanotubes can be electrically coupled through interposed gap-junction channels. Proc Natl Acad Sci U S A.

[43] Chauveau A, Aucher A, Eissmann P, Vivier E, Davis DM (2010). Membrane nanotubes facilitate long-distance interactions between natural killer cells and target cells. Proc Natl Acad Sci U S A.

[44] Ramírez-Weber Felipe-Andrés, Kornberg Thomas B (1999). Cytonemes. Cell.

[45] Gradilla A-C, Guerrero I (2013). Cytoneme-mediated cell-to-cell signaling during development. Cell Tissue Res.

[46] Mattila Pieta K., Lappalainen Pekka (2008). Filopodia: molecular architecture and cellular functions. Nature Reviews Molecular Cell Biology.

[47] Hervé J-C, Derangeon M (2013). Gap-junction-mediated cell-to-cell communication. Cell Tissue Res.

[48] Zani BG, Edelman ER (2010). Cellular bridges: routes for intercellular communication and cell migration. Commun Integr Biol.

[49] Abounit S, Zurzolo C (2012). Wiring through tunneling nanotubes--from electrical signals to organelle transfer. J Cell Sci.

[50] Davis DM, Sowinski S. Membrane nanotubes: dynamic long-distance connections between animal cells. nrm (2008). 9(6):431–6.

[51] Arkwright PD, Luchetti F, Tour J, Roberts C, Ayub R, Morales AP (2010). Fas stimulation of T lymphocytes promotes rapid intercellular exchange of death signals via membrane nanotubes. Cell Res.

[52] Hase K, Kimura S, Takatsu H, Ohmae M, Kawano S, Kitamura H (2009). M-sec promotes membrane nanotube formation by interacting with Ral and the exocyst complex. Nat Cell Biol.

[53] Schiller C., Diakopoulos K. N., Rohwedder I., Kremmer E., von Toerne C., Ueffing M., Weidle U. H., Ohno H., Weiss E. H. (2012). LST1 promotes the assembly of a molecular machinery responsible for tunneling nanotube formation. Journal of Cell Science.

[54] Kabaso D, Lokar M, Kralj-Iglič V, Veranič P, Iglič A (2011). Temperature and cholera toxin B are factors that influence formation of membrane nanotubes in RT4 and T24 urothelial cancer cell lines. Int J Nanomedicine.

[55] Lokar Maruša, Iglič Aleš, Veranič Peter (2010). Protruding membrane nanotubes: attachment of tubular protrusions to adjacent cells by several anchoring junctions. Protoplasma.

[56] Kimura S, Hase K, Ohno H (2013). The molecular basis of induction and formation of tunneling nanotubes. Cell Tissue Res.

[57] Pasquier Jennifer, Guerrouahen Bella S, Al Thawadi Hamda, Ghiabi Pegah, Maleki Mahtab, Abu-Kaoud Nadine, Jacob Arthur, Mirshahi Massoud, Galas Ludovic, Rafii Shahin, Le Foll Frank, Rafii Arash (2013). Preferential transfer of mitochondria from endothelial to cancer cells through tunneling nanotubes modulates chemoresistance. Journal of Translational Medicine.

[58] Lou E, Fujisawa S, Barlas A, Romin Y, Manova-Todorova K, Moore MAS (2012). Tunneling nanotubes: a new paradigm for studying intercellular communication and therapeutics in cancer. Commun Integr Biol.

[59] Lou E, Fujisawa S, Morozov A, Barlas A, Romin Y, Dogan Y (2012). Tunneling nanotubes provide a unique conduit for intercellular transfer of cellular contents in human malignant pleural mesothelioma. PLoS One.

[60] Koyanagi M, Brandes RP, Haendeler J, Zeiher AM, Dimmeler S (2005). Cell-to-cell connection of endothelial progenitor cells with cardiac myocytes by nanotubes: a novel mechanism for cell fate changes?. Circ Res.

[61] He K, Luo W, Zhang Y, Liu F, Liu D, Xu L (2010). Intercellular transportation of quantum dots mediated by membrane nanotubes. ACS Nano.

[62] Wang Z-G, Liu S-L, Tian Z-Q, Zhang Z-L, Tang H-W, Pang D-W (2012). Myosin-driven intercellular transportation of wheat germ agglutinin mediated by membrane nanotubes between human lung cancer cells. ACS Nano.

[63] Mi L, Xiong R, Zhang Y, Yang W, Chen J-Y, Wang P-N (2011). Microscopic observation of the intercellular transport of CdTe quantum dot aggregates through tunneling-nanotubes. JBNB.

[64] Rehberg M, Nekolla K, Sellner S, Praetner M, Mildner K, Zeuschner D (2016). Intercellular transport of Nanomaterials is mediated by membrane nanotubes in vivo. Small.

[65] Haimovich G, Ecker CM, Dunagin MC, Eggan E, Raj A, Gerst JE et al. Intercellular mRNA trafficking via membrane nanotubes in mammalian cells; 2017. ( vol 11). 10.1073/pnas.1706365114 PMC5699038 29078295

[66] Smith IF, Shuai J, Parker I (2011). Active generation and propagation of Ca2+ signals within tunneling membrane nanotubes. Biophys J.

[67] Bittins Margarethe, Wang Xiang (2017). TNT-Induced Phagocytosis: Tunneling Nanotubes Mediate the Transfer of Pro-Phagocytic Signals From Apoptotic to Viable Cells. Journal of Cellular Physiology.

[68] Gerdes H-H, Carvalho RN (2008). Intercellular transfer mediated by tunneling nanotubes. Curr Opin Cell Biol.

[69] Hurtig J, Chiu DT, Onfelt B (2010). Intercellular nanotubes: insights from imaging studies and beyond. Wiley Interdiscip Rev Nanomed Nanobiotechnol.

[70] Zhu D, Tan KS, Zhang X, Sun AY, Sun GY, Lee JC-M (2005). Hydrogen peroxide alters membrane and cytoskeleton properties and increases intercellular connections in astrocytes. J Cell Sci.

[71] Desir S, Dickson EL, Vogel RI, Thayanithy V, Wong P, Teoh D (2016). Tunneling nanotube formation is stimulated by hypoxia in ovarian cancer cells. Oncotarget.

[72] Osswald M, Jung E, Sahm F, Solecki G, Venkataramani V, Blaes J (2015). Brain tumour cells interconnect to a functional and resistant network. Nature.

[73] Reindl J, Shevtsov M, Dollinger G, Stangl S, Multhoff G (2019). Membrane Hsp70-supported cell-to-cell connections via tunneling nanotubes revealed by live-cell STED nanoscopy. Cell Stress Chaperones.

[74] Eugenin EA, Gaskill PJ, Berman JW (2009). Tunneling nanotubes (TNT) are induced by HIV-infection of macrophages: a potential mechanism for intercellular HIV trafficking. Cell Immunol.

[75] Rustom Amin (2016). The missing link: does tunnelling nanotube-based supercellularity provide a new understanding of chronic and lifestyle diseases?. Open Biology.

[76] Wang Y, Cui J, Sun X, Zhang Y (2011). Tunneling-nanotube development in astrocytes depends on p53 activation. Cell Death Differ.

[77] Riley T, Sontag E, Chen P, Levine A (2008). Transcriptional control of human p53-regulated genes. Nat Rev Mol Cell Biol.

[78] Pontes B, Viana NB, Campanati L, Farina M, Neto VM, Nussenzveig HM (2008). Structure and elastic properties of tunneling nanotubes. Eur Biophys J.

[79] Venkatesh VS, Lou E (2019). Tunnelling nanotubes: a bridge for heterogeneity in glioblastoma and a new therapeutic target?. Cancer Reports.

[80] Osswald M, Jung E, Wick W, Winkler F (2019). Tunneling nanotube-like structures in brain tumors. Cancer Reports.

[81] Desir S, O’Hare P, Vogel RI, Sperduto W, Sarkari A, Dickson EL, et al. Chemotherapy-induced tunneling nanotubes mediate intercellular drug efflux in pancreatic Cancer. Sci rep 2018; 8(1):1–13. Available from: URL. https://www.nature.com/articles/s41598-018-27649-x.pdf. 10.1038/s41598-018-27649-x PMC6013499 29930346

[82] Pasquier J, Galas L, Boulangé-Lecomte C, Rioult D, Bultelle F, Magal P (2012). Different modalities of intercellular membrane exchanges mediate cell-to-cell p-glycoprotein transfers in MCF-7 breast cancer cells. J Biol Chem.

[83] Thayanithy V, Dickson EL, Steer C, Subramanian S, Lou E (2014). Tumor-stromal cross talk: direct cell-to-cell transfer of oncogenic microRNAs via tunneling nanotubes. Transl Res.

[84] Antanavičiūtė I, Rysevaitė K, Liutkevičius V, Marandykina A, Rimkutė L, Sveikatienė R (2014). Long-distance communication between laryngeal carcinoma cells. PLoS One.

[85] Ady JW, Desir S, Thayanithy V, Vogel RI, Moreira AL, Downey RJ (2014). Intercellular communication in malignant pleural mesothelioma: properties of tunneling nanotubes. Front Physiol.

[86] Ady J, Thayanithy V, Mojica K, Wong P, Carson J, Rao P (2016). Tunneling nanotubes: an alternate route for propagation of the bystander effect following oncolytic viral infection. Mol Ther Oncolytics.

[87] Thayanithy V, Babatunde V, Dickson EL, Wong P, Oh S, Ke X (2014). Tumor exosomes induce tunneling nanotubes in lipid raft-enriched regions of human mesothelioma cells. Exp Cell Res.

[88] Bao L, Hazari S, Mehra S, Kaushal D, Moroz K, Dash S (2012). Increased expression of P-glycoprotein and doxorubicin chemoresistance of metastatic breast cancer is regulated by miR-298. Am J Pathol.

[89] Munoz JL, Bliss SA, Greco SJ, Ramkissoon SH, Ligon KL, Rameshwar P (2013). Delivery of functional anti-miR-9 by Mesenchymal stem cell-derived Exosomes to Glioblastoma Multiforme cells conferred Chemosensitivity. Mol Ther Nucleic Acids.

[90] Delaney G, Jacob S, Featherstone C, Barton M (2005). The role of radiotherapy in cancer treatment: estimating optimal utilization from a review of evidence-based clinical guidelines. Cancer.

[91] Baskar R, Lee KA, Yeo R, Yeoh K-W (2012). Cancer and radiation therapy: current advances and future directions. Int J Med Sci.

[92] Raychaudhuri B, Vogelbaum MA (2011). IL-8 is a mediator of NF-κB induced invasion by gliomas. J Neuro-Oncol.

[93] Krex D, Klink B, Hartmann C, von Deimling A, Pietsch T, Simon M (2007). Long-term survival with glioblastoma multiforme. Brain.

[94] Curran WJ, Scott CB, Weinstein AS, Martin LA, Nelson JS, Phillips TL (1993). Survival comparison of radiosurgery-eligible and -ineligible malignant glioma patients treated with hyperfractionated radiation therapy and carmustine: a report of radiation therapy oncology group 83-02. J Clin Oncol.

[95] Wank M, Schilling D, Reindl J, Meyer B, Gempt J, Motov S (2018). Evaluation of radiation-related invasion in primary patient-derived glioma cells and validation with established cell lines: impact of different radiation qualities with differing LET. J Neuro-Oncol.

[96] Rieken S, Habermehl D, Mohr A, Wuerth L, Lindel K, Weber K (2011). Targeting ανβ3 and ανβ5 inhibits photon-induced hypermigration of malignant glioma cells. Radiat Oncol.

[97] Pouget JP, Mather SJ (2001). General aspects of the cellular response to low- and high-LET radiation. Eur J Nucl Med.

[98] Baker M (2017). How the internet of cells has biologists buzzing. Nature.

[99] Winkler F, Wick W (2018). Harmful networks in the brain and beyond. Science.

[100] Lou E, Zhai E, Sarkari A, Desir S, Wong P, Iizuka Y (2018). Cellular and molecular networking within the ecosystem of Cancer cell communication via tunneling nanotubes. Front Cell Dev Biol.

[101] Takei H, Inaniwa T (2019). Effect of irradiation time on biological effectiveness and tumor control probability in proton therapy. Int J Radiat Oncol Biol Phys.

[102] Schulz-Ertner Daniela, Nikoghosyan Anna, Thilmann Christoph, Haberer Thomas, Jäkel Oliver, Karger Christian, Kraft Gerhard, Wannenmacher Michael, Debus Jürgen (2004). Results of carbon ion radiotherapy in 152 patients. International Journal of Radiation Oncology*Biology*Physics.

[103] Levin W P, Kooy H, Loeffler J S, DeLaney T F (2005). Proton beam therapy. British Journal of Cancer.

[104] Makishima H, Yasuda S, Isozaki Y, Kasuya G, Okada N, Miyazaki M (2019). Single fraction carbon ion radiotherapy for colorectal cancer liver metastasis: a dose escalation study. Cancer Sci.

[105] Fukumitsu Nobuyoshi, Sugahara Shinji, Nakayama Hidetsugu, Fukuda Kuniaki, Mizumoto Masashi, Abei Masato, Shoda Junichi, Thono Eriko, Tsuboi Koji, Tokuuye Koichi (2009). A Prospective Study of Hypofractionated Proton Beam Therapy for Patients With Hepatocellular Carcinoma. International Journal of Radiation Oncology*Biology*Physics.

[106] Sahu P, Jena SR, Samanta L (2018). Tunneling nanotubes: a versatile target for Cancer therapy. Curr Cancer Drug Targets.

[107] Osswald M, Solecki G, Wick W, Winkler F (2016). A malignant cellular network in gliomas: potential clinical implications. Neuro-oncology.

[108] Vignais M-L, Caicedo A, Brondello J-M, Jorgensen C (2017). Cell connections by tunneling nanotubes: effects of mitochondrial trafficking on target cell metabolism, homeostasis, and response to therapy. Stem Cells Int.

